# The Improvement of Food Supplies

**Published:** 1919-06-28

**Authors:** 


### THE IMPROVEMENT OF FOOD SUPPLIES.

If the war has taught us nothing else, it has at least compelled us to examine very carefully and critically the quality and quantity of our food supplies, and has resulted in a rehabilitation in politics of the long-neglected agricultural interests of the kingdom. How long the professional politician will pay attention to such topics, now that the war is over, depends first and foremost on the electorate. If the people of this country lose interest in the matter, it is certain that the expert vote-catchers will revert to the policy of careless neglect which was the rule before the war. If an instructed public opinion demands sincere attention to the amelioration of food supplies, both home and foreign, the electioneering instinct which sways the bulk of members of Parliament will see to it that improvement takes place. That there is room for such improvement has for years been dinned into the ears of an unresponsive public by all those best qualified to judge; chiefly, that is to say, by the medical profession, and especially by those members of it whose specialty is preventive medicine. The conditions of our milk supply, for example, have been denounced over and over again by all the most competent scientific authorities; but of constructive legislation to deal with the grave evils disclosed years ago there has been extremely little so far. Now, however, we have a Ministry of Health, and better things are hoped for. It must still be realised that the Ministry is a department of the Government of the day; that its head must resign when the Government resigns; and that his suggestions for action are liable and likely to be overruled by the Cabinet, not only if they threaten to be unpopular with sections of the electorate, but even if, however necessary they may be, they are crowded out of the sessional programme by measures regarded as more likely to secure votes for the next general election.

With this preliminary warning that the future of the national health remains, and must remain, the preoccupation primarily of the electorate, which has not discharged its duty merely by acquiescing in the formation of the Health Ministry, let it be acknowledged that at present a most commendable activity is manifest in the investigation of a number of the most pressing problems connected with the nation's food supply. First and foremost the Milk Control Board has issued a memorandum* which recapitulates many long-known and other recently elicited facts, of a most disquieting nature, concerning this staple element in the diet of the nation's children. The memorandum concludes with an outline of general policy which is thoroughly sound: incidentally it implicitly condemns the hitherto prevailing apathy of Parliament by stating certain proposals for improvement which "the Government should concern itself more closely" (with) "than it has hitherto done." The mere list of the measures recommended is a severe commentary on the negligence and omissions of the past. The Report of the Food Investigation Board for 1918 is also just published,† and reveals the existence and activities of a number of committees which have done good work in various special departments of the Board's varied and manifold task. The Report admits that the appointment of these committees was undertaken with reluctance, because research by committee is suspect: in a happy sentence they grant that "in the pursuit of science the instinct of the individual counts for more than the combined intelligence of the many." Notwithstanding this drawback, which it is pleasing to find that the Board is sensible of, it may be stated that the results have been already very valuable. Separate report deals with the design of railway wagons for the carriage of perishable foods, and with the problems of refrigeration. A Fish Preservation Committee, working under difficulties which are now to be surmounted, has been mainly occupied with the method of fish preservation known as "brine freezing," which promises results of great value. This method, we are told, was tried many years ago by a private firm, but "the time was not then ripe for its acceptance"; this phrase means, it may be supposed, that vested interests in the trade and the ignorance and apathy of the public would not allow of a fair trial; but for the war it might have been shelved for good and all, or until some of our rivals across the North Sea had obtained a monopoly of it. Oils and fats, fruits and vegetables, and canned foods are all the subjects of special committees, from whose labours scientific results of the greatest benefit will accrue to the people and the nation—on one condition, which is essential. That condition is that those to whom all ministries, governments, and members of Parliament are finally responsible, namely, the electorate at large, shall take an intelligent interest in a subject touching so vitally their health and their children's health: neither obstructing research properly conducted, nor allowing ignorant commercialism to prejudice the national health, nor putting executive power into the hands of fanatics and sentimentalists. And this means a better and more practical scheme of national education than the nation has hitherto had: for it cannot be disguised that in the present state of the mal-education of the democracy the condition postulated is little likely to be fulfilled.

* *Memorandum on the Milk Supply.* H.M. Stationery Office. Price 1d. net.

† H.M. Stationery Office. Price 3d. net.

